# Breast Cancer Heterogeneity

**DOI:** 10.3390/diagnostics11091555

**Published:** 2021-08-27

**Authors:** Caterina Fumagalli, Massimo Barberis

**Affiliations:** Division of Pathology, IEO, European Institute of Oncology IRCCS, 20141 Milan, Italy; massimo.barberis@ieo.it

**Keywords:** breast cancer, inter-tumor heterogeneity, intra-tumor heterogeneity

## Abstract

Breast tumor heterogeneity is a major challenge in the clinical management of breast cancer patients. Both inter-tumor and intra-tumor heterogeneity imply that each breast cancer (BC) could have different prognosis and would benefit from specific therapy. Breast cancer is a dynamic entity, changing during tumor progression and metastatization and this poses fundamental issues to the feasibility of a personalized medicine approach. The most effective therapeutic strategy for patients with recurrent disease should be assessed evaluating biopsies obtained from metastatic sites. Furthermore, the tumor progression and the treatment response should be strictly followed and radiogenomics and liquid biopsy might be valuable tools to assess BC heterogeneity in a non-invasive way.

## 1. Introduction

**Figure 1:**
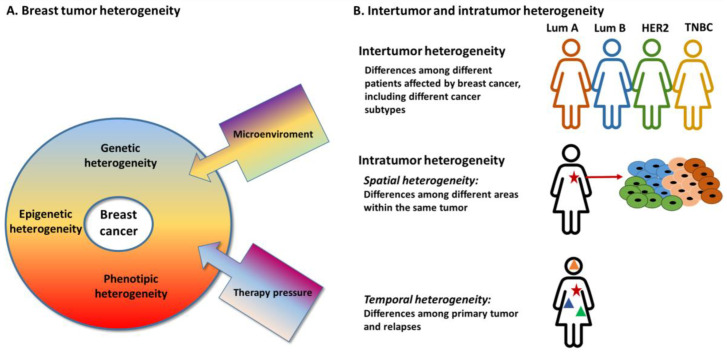
Breast tumor heterogeneity. (**A**) Factors that contribute to breast tumor heterogeneity; (**B**) Type of breast tumor heterogeneity: Inter-tumor and intra-tumor heterogeneity. Star: primary tumor, Triangles: relapses.

Breast cancer (BC) is the most common female malignancy [1] and, although great efforts have been made to develop effective treatment strategies, it still remains the leading cause of tumor-related mortality in women worldwide. Breast cancer is typically a highly heterogeneous disease. Breast tumor heterogeneity has been observed and described since the nineteenth century [2] and these differences have served as the basis for disease classification. Hawkins et al. in 1988 reported variations in estrogen receptor concentration due to variations in tissue cellularity [3]. Breast tumor heterogeneity was observed among different patients (inter-tumor heterogeneity) and even within each individual tumor (intra-tumor heterogeneity), occurring as spatial and temporal heterogeneity [4] (Figure 1). The spatial heterogeneity involves distinct areas within a tumor that have differences at the phenotypic, transcriptomic, epigenetic and genomic levels; the temporal heterogeneity refers to variations occurring over time during tumor progression between primary and metastasis or among different metastatic lesions (intra-metastatic heterogeneity) [5]. Indeed, the tumor evolution itself as well as the therapy-induced clonal selection could result in intra-tumor or intra-metastases heterogeneity. In this scenario, even the microenvironment, equally affected by spatial heterogeneity phenomena, could boost the tumor heterogeneity. Actually the interactions between BC and stromal cells, the presence of chronic inflammation, the levels of nitric oxide and reactive oxygen species secretion and the extracellular matrix, as scaffold for protein and cell-specific trafficking, are some of the factors that could influence tumor development and progression [6,7].

## 2. Inter-Tumor Heterogeneity

Inter-tumor heterogeneity reflects differences observed among different patients with BC. Traditionally, inter-tumor heterogeneity has been associated with different BC histological subtypes, treatment sensitivity response, and clinical outcomes. According to estrogen and progesterone receptors (ER and PR) status, Ki67 proliferation index and HER2 expressions, BC are grouped in Luminal-A (ER^+^ and/or PR^+^, Ki67 < 20, HER2^−^), Luminal B- (ER^+^ and/or PR^+^, Ki67 ≥ 20, HER2^−^), Luminal B^+^ (ER^+^ and/or PR^+^, HER2^+^), HER2^+^ non-Luminal (ER^−^, PR^−^, HER2^+^), and triple-negative (ER^−^, PR^−^, HER2^−^). These BC subgroups have different prognosis and specific therapy response. In detail, luminal A and luminal B are the prevalent BC subtypes and have been associated with a better outcome [8], whereas an aggressive course has been reported for triple negative tumors [9]. Moreover, the presence of ER or HER2 positivity leads to endocrine therapy benefit or target-therapy sensitivity, respectively [10,11], while immunotherapy treatments have been proposed for triple negative tumors [12,13]. In this scenario, immunogenic biomarkers including tumor mutational burden (TMB), immune gene expression and mismatch repair deficiency (MMRd) are of paramount importance for selecting who could more likely benefit from immunotherapy regimens. The different BC subtypes show different biomarkers prevalence. In detail, hormone receptor (HR) negative breast cancers are characterized by a higher TMB, immune gene expression and MMRd frequency compared with HR positive tumors. Instead, HER2 negative BCs have a lower TMB and downregulation of immune gene expression compared to HER2 positive BCs. These results suggest that HR negative and HER2-positive tumors may be more likely to benefit from immunotherapies [14].

In addition, each BC subtype has distinctive molecular hallmarks [15]. Next-generation sequencing (NGS) analysis of primary breast tumors revealed that very few cancer-related genes are mutated at high frequency, altered in more than 10% of all BC subtypes, namely *GATA3*, *TP53* and *PIK3CA*. However, a high inter-tumor heterogeneity has been observed. In detail, *TP53* mutations were more common in triple negative tumors, whereas *PIK3CA* mutations were more frequent in Luminal/HER2 negative and HER2 positive tumors [16]. Besides, peculiar genetic aberrations are associated with specific BC subtypes, as *CDH1* mutations in Luminal A tumors, *CCND1*, *FGF3*, *FGF19*, and *FGFR1* copy gains in estrogen receptor positive BC, whereas *MYC* amplification, *NF1* mutations and *BRCA1/2* pathogenic variants were recurrent in triple negative tumors [16,17,18,19]. 

Table 1 summarizes the main epidemiological, phenotypical and genetic characteristics of BC subtypes.

The identification of peculiar alterations can also be associated with prognosis or response to therapy. For example, the detection of *FGFR1* amplification has been associated with an increased risk of late recurrences in ER-positive BC [20], or *PIK3CA* mutations have been considered predictive of neoadjuvant chemotherapy resistance in HER2 positive BCs [16]. Furthermore, different pathological features can result in different prognostic value according to different BC subtypes. The presence of TILs has a prognostic significance in HER2 positive and triple negative breast cancers where a higher stromal lymphocytic infiltration has been associated with better prognosis, but this was not confirmed in luminal tumors [21,22,23].

## 3. Intra-Tumor Heterogeneity

Intra-tumor heterogeneity refers to the heterogeneity seen between different regions of a primary tumor, between a primary tumor and a metastatic lesion, or among different metastatic lesions. Indeed, differences in the tumor genome, epigenome, transcriptome and proteome could be revealed in different phenotypes, creating diagnostic and therapeutic challenges. In addition, heterogeneity of phenotypes within the same tumor can be influenced by the surrounding microenvironment. The extracellular matrix (ECM) is the major component of the local microenvironment—niche—of cancer cells. The ECM is a complex network of macromolecules such as proteins, proteoglycans, and polysaccharides with different physical, biochemical, and biomechanical properties. There is a bidirectional communication between tumor cells and the ECM through cell-matrix interactions resulting in a dynamic remodeling of ECM. The deregulation of ECM remodeling, including changes in the three-dimensional spatial organization of ECM and of its biochemical and physical properties, can create a microenvironment that promotes tumorigenesis and metastasis [24,25]. Indeed, stromal desmoplasia, characterized by excessive deposition of fibrillary collagen in the surroundings of the tumor, is very common in BC. Bundles of straightened and aligned collagen fibers perpendicularly oriented to the tumor boundary are supportive of invasive tumor growth [26]. Organoid models may offer the possibility of investigating in vitro the tumor microenvironment, capturing functional parameters related to tumor heterogeneity [27]. However, these models should be used with caution because they cannot reflect the complexity of the dialogue among the components of microenvironment in vivo. Recent advances in transcriptomic and proteomic studies at a single cell level could probably offer new insight in this field [25,28].

### 3.1. Spatial Heterogeneity

Spatial heterogeneity refers to heterogeneity occurring among different geographical regions of the same tumor.

Morphologically distinct regions of a tumor can exhibit different mutational landscapes, associated with distinct genetic aberrations [29,30]. The sub-clonal structure of primary BC is of paramount importance for tumor evolution and therapy response. Nik-Zainal et al. performed whole-genome sequencing analyses coupled with bioinformatic algorithms for the phylogenetic tree design across 21 primary breast tumors of different subtypes and revealed that several subclones were present in each tumor, harboring different somatic mutations, copy-number aberrations, and chromosomal rearrangements [31,32]. Moreover, Yates reported that potentially targetable mutations were sub-clonal and markers of disease progression arose within detectable subclones of antecedent lesions [33]. Interestingly, the sub-clonal architecture may develop in a nonrandom way for a tumor cell contact-mediated mechanism that could induce clonal expansion of specific subclones, as recently described in association with HER2:PIK3CA mutated clonal dynamics and fibronectin expression [34].

In addition, the intra-tumor heterogeneity has also been correlated to different immune microenvironments. In the same tumor, a heterogeneous pattern of Tumor-Infiltrating Lymphocytes (TILs) can be observed, with different composition of immune cell subpopulations [35,36,37]. Lymphocytes have a highly mobile nature and can lead to rapidly changing spatial heterogeneity, resulting in immunologically active or silent niches, crucial for response to immunotherapy. Moreover, a heterogeneous immunohistochemical expression of MMR protein has been recently reported in more than 12% of BCs [38]. The relative frequency of heterogeneous protein expression ranged from 33% for PMS2 to 50% for MSH6, with higher rates in luminal tumors. Even the PD-L1 expression exhibits spatial heterogeneity in both the tumor cells and the infiltrating immune cells or node lymphocytes in primary tumors and lymph nodal metastases [39]. All these findings suggest that the analysis of a single area of the tumor may not represent the status of the whole breast tumor. Therefore, even the gene signatures assays such as Oncotype or Mammaprint, valuable tools for recurrence prediction and treatment decision making, may be influenced by tumor heterogeneity. As highlighted in the study of Gyanchandani et al., in highly heterogeneous tumors the evaluation of a single tumor core could under- or overestimate the recurrence risk. In this scenario, the analysis of the most heterogeneous sections or the assessment of multiple core of the same BC, reflecting the intra-tumor heterogeneity, could be effective strategies to apply for the performance of clinically useful gene expression panels [40].

The levels of uniformity or heterogeneity of specific markers within the same tumor have an important clinical value as they have been recently associated to prognosis and therapy response. *ERBB2* gene amplification assessed by fluorescence in situ hybridization (FISH) or HER2 protein overexpression assessed by immunohistochemistry are the main primary predictors of responsiveness to HER2-targeted therapies in BC. In HER2 positive BC, a heterogeneous HER2 immunohistochemical staining and *ERBB2* gene amplification evaluated by FISH have been associated with shorter survival and tumor progression [41,42,43]. Moreover, in HER2 positive BC, the response to HER2-targeted therapy could be modulated by the level of HER2 heterogeneity, defined as an area with ERBB2 amplification in >5% but <50% of tumor cells, or a HER2 negative area by FISH. Patients with a higher level of heterogeneity are less likely to have a complete pathologic response to neoadjuvant pertuzumab and T-DM1 treatment [44]. Even the response to endocrine therapy is related to the level and uniformity of ER expression: tumors strongly expressing ER in the majority of tumor cells show a better outcome than ER-heterogeneous tumors [45,46] whereas intra-metastatic ER heterogeneity has been reported as an independent predictor of poor patient survival [47].

### 3.2. Temporal Heterogeneity

Temporal heterogeneity refers to the evolution of a tumor over time, including the transition from in situ breast carcinoma to invasive cancer and to metastatic disease. The tumor is a dynamic entity that can change during progression. The tumor modeling is a result of additional genetic alterations acquired during cancer progression but also as a consequence of treatments in selecting therapy resistant clones, or influenced by the tumor-stroma-immune system interactions [48].

The most relevant evidence of temporal heterogeneity is the discordance in terms of ER, PR and HER2 expression that could emerge between primary tumors and their matched metastatic lesions. Discordant expression rates can be observed in a not irrelevant quota of patients and varies from 9–30% for ER, 15–45% for PR and 4–16% for HER2 expressions, and can affect tumor behavior and treatment response [49,50,51,52,53,54,55,56,57]. According to PAM50 gene expression, breast primary tumors shift toward unfavorable subtypes in metastases, with a worse prognosis associated with a change from luminal primary tumor to non-luminal distant metastasis [58]. The status of ER, PR, HER2 and Ki-67 expression can also change following neoadjuvant treatment (NAT). The biomarker status change after NAT is common, affecting approximately 30% of patients and has prognostic value [59,60]. In particular, no biomarker change is associated with improved survival whereas the shift from HR^+^/HER2^−^ to HR^−^/HER2^−^ is associated with worse prognosis [59,60]. Consequently, the retesting of hormone receptors and HER2 status after NAT and at relapse may be useful both for prognosis and treatment purposes.

Another observation is the PD-L1 immunohistochemical staining discrepancy between primary tumors and metastases. A recent meta-analysis indicates that pooled PD-L1 positivity rate, considering both tumor and immune cells, was higher in primary breast tumors compared to metastases. However, PD-L1 discordance between primary BC and metastasis was bi-directional and was more common when PD-L1 expression was assessed in immune cells only [61].

Recent NGS analyses evaluating paired primary tumors and matched metastatic lesions confirm the molecular heterogeneity underpinning metastatic progression [17,62,63,64,65]. Interestingly, a quota of mutations arisen during the metastatic process could be targeted by biological drugs (e.g., affecting ERBB2, BRCA2, PIK3CA) [66]. In our recent study we evaluated primary tumors and matched relapses of 61 patients affected by BC. We detected in 39 (63.9%) cases additional private alterations in the relapse samples only, including 12 (19.7%) of patients with potentially actionable aberrations [17]. Moreover, the extent of temporal intra-tumor heterogeneity detected might be proportional to the time elapsed between the diagnosis of the primary tumor and the occurrence of metastatic relapse, as the longer the time, the higher the extent of heterogeneity [17,65].

Primary cancers have more clonal variability in terms of mutations and structural modifications than their metastatic counterparts, suggesting that therapy may lead to clonal selection. On the other hand, the therapy could induce molecular changes emerging during/after treatments as the enrichment in MCL1, JAK2, CDK6/CCND1–3 gene copy number gain detected by massive parallel sequencing and PTEN deletions and/or mutations in the post-neoadjuvant-chemotherapy setting of TNBC [67]. Patients with BRCA1/2 alterations treated with poly(ADP) ribose polymerase (PARP) inhibitors and platinum-based chemotherapy could develop resistance as a result of secondary mutations in BRCA1/2 genes [68]. Moreover, ESR1 mutation onset in recurrent disease of luminal primary tumors treated with endocrine therapies is a marker of endocrine therapy resistance and disease progression. The infrequent ESR1 alterations found in treatment-naive tumors suggest that the selective pressure of treatment may lead to clonal expansion of rare mutant clones [69,70,71].

A pivotal contribution to understanding the dynamic changes occurring during BC progression has been recently added by the molecular screening initiative—AURORA program—of the Breast International Group. The AURORA program aimed to evaluate the processes of relapse in metastatic breast cancer, performing analysis of the genomic and transcriptomic profile of paired primary BC and metastases of 381 patients. They identified genomic alterations enriched in metastases namely ESR1, PTEN, CDH1, PIK3CA and RB1 mutations, MDM4 and MYC gene copy number gains, and ARID1A deletions. Moreover, ESMO Scale of Clinical Actionability for molecular Targets (ESCAT) tier I/II alterations were detected in over half of the patients, with crucial impact for target therapy selection; on the other hand, metastases had lower immune score and an increased number of immune permissive cells [62].

All these findings highlight the importance of evaluating the metastatic disease for planning the most effective—single patient tailored—therapeutic strategy.

## 4. Clinical Implications of Tumor Heterogeneity

Tumor heterogeneity poses important clinical challenges, both for prognosis and response to therapy. The identification of intra-tumor heterogeneity itself, whether within the primary tumors or between primary tumor and metastatic counterparts or intra-metastatic, is a hallmark of malignancy, linked to poor patient outcomes [72,73,74]. From a therapeutic point of view, inter-tumor heterogeneity implies that each breast cancer can be different in every patient, foreclosing ‘one-size-fits-all’ treatment approaches. In addition, the presence of different tumoral clones within an individual tumor adds more difficulties for the selection of the most effective therapy [75]. Indeed, intra-tumor heterogeneity entails that targeting the predominant aberrations might not be effective against all the tumoral clones, but the selective pressure of anticancer treatments may lead to the expansion of different resistant subclones [72,73,74]. Patient-derived tumor xenograft (PDTXs) can be useful models for mirroring the BC inter- and intra-tumor heterogeneity and can be adopted for in vitro drug screening and in vivo predicting drug response [76,77]. Recently Georgopoulou et al. applied a single-cell breast cancer mass cytometry (BCMC) panel in a biobank of PDTX models and showed that different tumor cell phenotypes with distinct drug sensitivities can coexist in the same tumors and display different dynamics under therapy [78].

Currently tumor heterogeneity is still not commonly evaluated in clinical practice, as the major issue is how to assess it. In the primary setting, the combination of digital pathology with automated image analysis of the whole tumor tissue section could be a useful tool to capture intra-tumor heterogeneity [79,80] but in the metastatic setting this is more challenging. Evaluating the genomic landscape of cancer through a single tumor biopsy gives a spatial- and temporal snapshot of the disease. On the contrary, liquid biopsy, including circulating tumor DNA (ctDNA) and circulating tumor cells (CTC), could be more representative of the temporal and spatial heterogeneity of the breast cancer genome [81]. The liquid biopsy approach could be useful not only for the repeatability over time, essential for the monitoring of tumor progression, but also because the circulating tumor biomarkers may derive from clones with higher metastatic potential that should be targeted [82,83,84]. A proof of liquid biopsy’s usefulness in the management of BC patients is the detection of ESR1 mutations in ctDNA obtained from plasma of ER-positive metastatic BC patients, a biomarker of aromatase inhibitor therapy resistance [85].

Another non-invasive and promising approach, rapidly expanding in recent years, is the use of radiomics. Radiomics can extract high-throughput quantitative data from non-invasive radiological images, improving cancer diagnosis and treatment [86,87,88]. The radiogenomics correlates radiomic features and genomic characteristics, with the goal of identifying clinically relevant molecular alterations associated with outcomes. Compared to the traditional biopsy of metastatic lesion approach, radiogenomic analyses are non-invasive and easily repeatable and may become a surrogate for tissue-based genomic analyses. Moreover, the radiogenomics analysis can capture intra-tumor spatial heterogeneity, assuming a clinically relevant prognostic value. Indeed, in a recent study the radio-genomic signatures of imaging scale heterogeneity were used to classify patients affected by breast cancer into groups with distinct subclone compositions, linked to prognosis [89].

## 5. Conclusions

Breast cancer is widely characterized by phenomena of heterogeneity. Inter-tumor and intra-tumor heterogeneity, spatial and temporal differences concerning phenotypic and genotypic aspects, have a clear impact on the clinical management of BC patients, affecting prognosis and therapy response. The tumor genetic landscape could change during tumor progression, hence the need to evaluate metastatic biopsies to assess the best—i.e., single patient tailored—therapeutic strategy for patients with recurrent disease. Moreover, the treatment response or the onset of resistance mechanisms should be strictly followed for a tuning of the powerful therapeutic strategy as a combination of agents that target different driver mutations. In this scenario, radiogenomics and liquid biopsy tools might be the future, having the potential to capture BC heterogeneity in a non-invasive way.

## Figures and Tables

**Table 1:** Different epidemiological, phenotypical and genetic characteristics among BC subtypes.

	Luminal-A	Luminal-B	HER2^+^ Non-Luminal	Triple Negative
Hormone receptors	ER^+^ and/or PR^+^	ER^+^ and/or PR^+^	ER^−^, PR^−^	ER^−^, PR^−^
HER2	HER2^−^	HER2^+^/^−^	HER2^+^	HER2^−^
Ki67	Ki67 < 20	Ki67 ≥ 20		
Prevalence among populations [15]	Non Africans 47–61%	All 8–18%	Asians 19–36%	Africans 27–37%
	Africans 26–27%		Caucasians 13.7–19%	Others 8–20%
			Africans 15–23%	
Most frequently/peculiar mutated genes [19]	PIK3CA (47.5%)	TP53 (36.0%)	TP53 (70.5%)	TP53 (89.5%)
	CDH1 (21.8%)	PIK3CA (29.9%)	PIK3CA (33.3%)	TTN (24.0%)
	GATA3 (15.4%)	TTN (20.3%)	TTN (28.6%)	BRCA1 (7.6%)
	MAP3K1 (14.2%)	GATA3 (20.3%)	MUC1 (19.2%)	NF1 (4.7%)
Most frequently/peculiar CNA [19]	CCND1 (17.0%)	CCND1 (25.9%)	ERBB2 (70.5%)	MYC (35.7%)
	FGF19 (16.8%)	FGF4 (24.4%)		
	FGF3 (16.6%)	FGF3 (23.9%)		
	FGF4 (16.6%)	FGFR1 (19.3%)		
Biologic pathway [15]	ER signaling	ER signaling	HER-2 signaling	Immune response
	ECM	ECM	Proliferation	ECM
		Proliferation	Immune response	Proliferation
			Tumor invasion	

CNA: Copy number alterations, ECM: Extracellular matrix.

## Data Availability

Not applicable.
